# Induced Pluripotent Stem Cells to Model and Treat Neurogenetic Disorders

**DOI:** 10.1155/2012/346053

**Published:** 2012-07-19

**Authors:** Hansen Wang, Laurie C. Doering

**Affiliations:** ^1^Faculty of Medicine, University of Toronto, 1 King's College Circle, Toronto, ON, Canada M5S 1A8; ^2^Department of Pathology and Molecular Medicine, Faculty of Health Sciences, McMaster University, HSC 1R1, 1280 Main Street West, Hamilton, ON, Canada L8S 4K1

## Abstract

Remarkable advances in cellular reprogramming have made it possible to generate pluripotent stem cells from somatic cells, such as fibroblasts obtained from human skin biopsies. As a result, human diseases can now be investigated in relevant cell populations derived from induced pluripotent stem cells (iPSCs) of patients. The rapid growth of iPSC technology has turned these cells into multipurpose basic and clinical research tools. In this paper, we highlight the roles of iPSC technology that are helping us to understand and potentially treat neurological diseases. Recent studies using iPSCs to model various neurogenetic disorders are summarized, and we discuss the therapeutic implications of iPSCs, including drug screening and cell therapy for neurogenetic disorders. Although iPSCs have been used in animal models with promising results to treat neurogenetic disorders, there are still many issues associated with reprogramming that must be addressed before iPSC technology can be fully exploited with translation to the clinic.

## 1. Introduction

Most of our current knowledge about the central nervous system (CNS) and neural function in patients with neurological diseases has been obtained from postmortem tissues that often represent the end stage of the disease. The inability to sample live CNS tissues impedes our progress to understand aspects of the neuropathological abnormalities that develop during the course of the disease [1–4]. Animal models can mimic genetic forms of human neurological diseases, and our understanding of the mechanisms of neurological diseases has been significantly advanced with transgenic/knockout technologies. However, these technologies are mainly limited to monogenetic disorders and thus only represent a minority of diseases. Additionally, in many cases of neurological disorders with a defined causal gene(s), modeling with animal transgenic technology is inadequate due to species differences, genetic backgrounds, or other technical challenges [3, 5, 6]. More strikingly, numerous candidate drugs with promise in animal model screening have failed when translated to human clinical trials. The failure of translation to the clinic centers on the complexity of the human brain and the difficulty to model disease specific phenotypes in nonhuman systems [5]. This situation indicates that an advancement towards more human relevant models is definitely needed to accurately study neurogenetic disorders.

The elegant and seminal work by Takahashi and Yamanaka showed that retroviral expression of a set of four genes (Oct4, Sox2, Klf4, and c-Myc) can convert somatic cells into a pluripotent state [7, 8]. Like other pluripotent stem cells, induced pluripotent stem cells (iPSCs) can be coaxed to differentiate into neurons and glial cells, as well as other terminally differentiated cell types by exposure to a combination of growth factors and cell culture conditions [9, 10]. Therefore, human iPSCs make it possible to study human CNS neuronal lineages. Neurons derived from iPSCs carry the genetic information from patients with a specific mutation or a neurological disease [3, 11, 12]. Over the past few years, the progress in cell reprogramming has accelerated the generation of iPSCs, and iPSCs have now been derived from several easily accessible human cell types, including blood cells, keratinocytes, and dermal fibroblasts [4, 5, 13–15]. The iPSC technology has opened new windows for modeling human diseases, identifying therapeutic targets, developing drug screening systems, and providing continuous autologous cell sources with potential for cell therapies [1, 5, 11, 15–20].

Here, the recent efforts and key findings when using iPSCs to model neurogenetic disorders are reviewed. The potential of iPSCs from patients as platforms for drug screening and as a source for cell therapy are presented. Additionally, some of the challenges in iPSC modeling and in iPSC based therapy of neurogenetic disorders are highlighted.

## 2. iPSCs for Modeling Neurogenetic Disorders

### 2.1. Genetic Neurodevelopmental Disorders

Genetic neurodevelopmental disorders include a wide range of diseases characterized by impairment of neuronal function during development. These conditions are monogenic or multigenic. Disease-specific iPSC lines have been generated from patients with neurodevelopmental diseases including Rett syndrome, Fragile X syndrome, Down syndrome, Angelman syndrome, Prader-Willi syndrome, and Timothy syndrome (Table 1). The iPSC based models of neurodevelopmental disorders recapitulate the early steps in neural development within genetic backgrounds that are linked to the specific disorder and may help to identify the underlying cellular and molecular mechanisms and establish novel therapeutics.

#### 2.1.1. Rett Syndrome

Rett syndrome (RTT) is a neurodevelopmental autism spectrum disorder, caused by mutations in the methyl CpG-binding protein (*MECP2*) gene [21–25]. The iPSC derived neurons from patients with RTT reveal relevant neuronal phenotypes, such as neuronal maturation defects [26–28]. Consistent with RTT animal models and human postmortem brain tissues [29], the cell soma size of RTT neurons is decreased in comparison with nonaffected controls. Additionally, neurons derived from RTT iPSCs have fewer synapses, reduced spine densities, altered calcium signaling, and electrophysiological defects, suggesting a communication problem in RTT neuronal networks [27]. Treatment with insulin growth factor 1, a growth factor known to ameliorate the phenotype of RTT mice, improves the RTT iPSC-neuronal phenotypes, indicating that synaptic defects can be rescued in neurons derived from RTT patients [27, 30]. The iPSCs can be directed to produce glutamatergic neurons that generate action potentials and form functional excitatory synapses. A recent study found that iPSC derived neurons from heterozygous *MECP*2^308^ mice showed defects in the generation of evoked action potentials and in glutamatergic synaptic transmission [31], as previously reported in brain slices [32–34]. These MeCP2 deficient neurons fired fewer action potentials and displayed decreased action potential amplitudes, diminished peak inward currents, and higher input resistance relative to wild-type iPSC derived neurons, suggesting that disturbed sodium channel function may contribute to the dysfunctional RTT neuronal network. These phenotypes were further confirmed in neurons derived from independent wild-type and hemizygous mutant iPSC lines, indicating that these reproducible deficits are attributable to the MeCP2 deficiency [31]. Taken together, these studies demonstrate that RTT iPSC derived neurons recapitulate deficits observed previously in primary neurons, and these identified phenotypes further indicate the requirement of MeCP2 in neuronal development and the maintenance of normal brain function.

MeCP2 is a protein involved in global DNA methylation [35–38]. The activity of L1 retrotransposons during brain development can have an impact on gene expression and neuronal function. L1 neuronal transcription and retrotransposition in rodents are increased in the absence of MeCP2 [39–41]. Studies with neuronal progenitor cells derived from human iPSCs revealed that patients with RTT, carrying MeCP2 mutations, have increased susceptibility for L1 retrotransposition, suggesting a new potential molecular mechanism underlying RTT [42]. *MECP2* is an X-linked gene subject to random X chromosome inactivation (XCI) resulting in mosaic expression of mutant *MECP2*. The XCI status of RTT iPSCs has been inconsistent among different studies. Some reported RTT iPSCs in which the inactive X chromosome of the founder somatic cell is reactivated. Conversely, others reported that RTT iPSCs retain the inactive X chromosome of the founder somatic cell [43]. Marchetto et al. [27] found that RTT patients' iPSCs are able to undergo XCI. Cheung et al. [26] reported that iPSCs from classic female RTT patients with a functionally null mutation retained the *MECP2* mutation and an inactive X-chromosome in a non-random pattern. By taking advantage of the nonrandom pattern of XCI, they generated a pair of isogenic wild-type and mutant *MECP2* expressing RTT iPSC lines that retain the *MECP2* expression pattern upon differentiation into neurons. The mutant RTT iPSC derived neurons showed a reduced soma size compared with the isogenic control RTT iPSC derived neurons. Kim et al. [28] found that some iPSCs could maintain XCI, whereas in others the X chromosome was reactivated. They isolated iPSCs that retained a single active X chromosome expressing either mutant or wild-type MeCP2, as well as iPSCs with reactivated X chromosomes expressing both mutant and wild-type MeCP2. Consistent with RTT phenotypes, the mutant monoallelic or biallelic RTT iPSC derived neurons also showed maturation defects. Thus, the isogenic control and mutant RTT iPSC lines represent an additional promising source for investigating the pathogenesis of RTT and the role of *MECP2* in human neurons.

Classic RTT is caused by mutations in the *MECP2* gene, whereas variants could be due to mutations in *CDKL5*. Mutations in *CDKL5* have been identified both in females with the early onset seizure variant of RTT and in males with X-linked epileptic encephalopathy [44–47]. CDKL5 is a kinase protein highly expressed in neurons, but its exact function inside the cell is largely unknown [46, 48, 49]. By using iPSCs derived from fibroblasts of patients with *CDKL5* mutations, Amenduni et al. [50] demonstrated that female *CDKL5* mutated iPSCs maintain XCI and clones express either the mutant *CDKL5* allele or the wild-type allele that serve as an ideal experimental control. Furthermore, these iPSCs can be differentiated into neurons and are suitable to model the pathogenesis of *CDKL5* related disorders.

#### 2.1.2. Fragile X Syndrome

Fragile X syndrome (FXS) is the most common inherited form mental impairment that is caused by an expanded CGG trinucleotide repeat in the 5′ untranslated region of the fragile X mental retardation (*FMR1*) gene leading to gene silencing and loss of the fragile X mental retardation protein (FMRP) [51–55]. In twenty years since the identification of the *FMR1* gene, numerous efforts have focused on understanding the consequences of loss of FMRP on neuronal development and function [51–54, 56–64]. The advent of iPSCs provides an option to study FXS in human models and allows investigations into aspects of FXS that are difficult to study in animal models. Human embryonic stem cell lines derived from embryos diagnosed with a full mutation showed that *FMR1 *is unmethylated when expressed in these cells, and *FMR1* gene silencing occurs only upon differentiation of embryonic stem cells [65, 66]. In contrast, for iPSCs generated from the fibroblasts of FXS patients, *FMR1 *remains methylated and transcriptionally silenced with the reprogramming process failing to reverse the methylation state of *FMR1* [66]. It seems that the current FXS iPSCs may not be suitable to model the effects of *FMR1* silencing during neuronal differentiation. A subsequent study further characterized the differentiation of FXS iPSCs into postmitotic neurons and glia [67]. In this study, the iPSC lines were generated from multiple patients with FXS. The authors found that clones from reprogrammed FXS patient fibroblast lines exhibit variation with respect to the predominant CGG repeat length in the *FMR*1 gene. In two cases, iPSC clones contained predominant CGG repeat lengths that were shorter than the corresponding input population of fibroblasts. In another case, reprogramming a mosaic patient having both normal and premutation length CGG repeats resulted in genetically matched iPSC clonal lines differing in *FMR1* promoter CpG methylation and FMRP expression. Using this panel of patient-specific FXS iPSC models, the authors demonstrated that aberrant neuronal differentiation from FXS iPSCs is correlated with the epigenetic modification of the *FMR1* gene and a loss of FMRP expression [67]. These findings provide evidence for roles of FMRP in early neurodevelopment prior to synaptogenesis and show potential for modeling FXS with iPSC technology.

#### 2.1.3. Down Syndrome

Down syndrome (DS) is neurodevelopmental disorder caused by trisomy of chromosome 21 [68–72]. Considering that mice do not have chromosome 21, it seems unlikely to completely recapitulate the disease features in mouse DS models. However, human neural progenitor cell (NPC) lines have been generated to model DS [73], and the iPSC models provide scientists with an additional approach to study underlying mechanisms [74]. Adults with DS develop early-onset Alzheimer's disease (AD), probably due to increased expression of a gene on chromosome 21 that encodes the amyloid precursor protein (APP) [75–77]. A recent study found that cortical neurons generated from iPSCs of DS patients could develop AD pathologies over months in culture, rather than years *in vivo*. These cortical neurons processed APP resulting in secretion of the pathogenic peptide amyloid *β*42, which formed insoluble amyloid aggregates. The gamma-secretase inhibitor could block the production of amyloid *β*42. Additionally, hyperphosphorylated tau protein, a pathological marker for AD, was found to be localized to cell bodies and dendrites of AD iPSC derived cortical neurons, which mimics the phenotypes of later stages of AD [78]. The generation of iPSC lines to investigate other similar defects, such as the trisomy of other chromosomes may be rewarding.

#### 2.1.4. Angelman Syndrome

Angelman syndrome (AS) is a neurodevelopmental disorder associated with genomic imprinting which results from a loss of function of the ubiquitin protein ligase E3A (*UBE3A*) gene [79–82]. Although iPSCs present an invaluable approach to modeling human disease, their usefulness could be limited in AS if the genomic imprinting marks are disturbed by the nuclear reprogramming of somatic cells to pluripotent stem cells. However, Chamberlain et al. [83] found that genomic imprinting was retained in AS iPSCs following nuclear reprogramming. The imprinting of *UBE3A* could be established during neuronal differentiation of AS iPSCs like normal brain tissue. In this case the paternal *UBE3A* allele was repressed in parallel to upregulation of the *UBE3A* antisense transcript. In addition, electrophysiological recordings detected AMPA-receptor-mediated spontaneous activity in mature neurons derived from AS iPSCs, indicating that functional neurons can be generated from AS iPSCs. The iPSC models will be further utilized to investigate the events related to AS, such as the developmental timing and mechanism of *UBE3A* repression in human neurons.

#### 2.1.5. Prader-Willi Syndrome

Prader-Willi syndrome (PWS) is a neurological genomic imprinting disorder, characterized by the lack of gene expression in the paternal chromosome region 15q11-q13, while the same region in the maternal chromosome is repressed by means of DNA methylation [84–86]. Chamberlain et al. [83] found that the PWS iPSC lines show no disrupted methylation patterns in the “Prader-Willi syndrome imprinting center” (PWS-IC) in comparison to the source fibroblast cell lines. This study indicated that similar to AS, genomic imprinting of PWS can be refractory to the epigenetic erasure produced during reprogramming. Another study by Yang et al. [87] further confirmed that PWS iPSCs retain a high level of DNA methylation in the imprinting center of the maternal allele and show concomitant reduced expression of the disease-associated small nucleolar RNA HBII-85/SNORD116 [87]. Moreover, these iPSCs could readily differentiate into tissues of the three germ layers, including neurons [87]. These studies indicate that iPSCs can be used to model genomic imprinting diseases.

#### 2.1.6. Timothy Syndrome

Timothy syndrome (TS) is a neurodevelopmental disorder caused by a missense mutation in the L-type calcium channel Ca_v_1.2 that is associated with developmental delay and autism [88–90]. Paşca et al. [91] generated cortical neuronal precursor cells and neurons from iPSCs derived from patients with TS. The authors found that the cells from these individuals have defects in calcium Ca^2+^ signaling and activity-dependent gene expression. The cells also showed abnormalities in differentiation, including decreased expression of the genes that are expressed in lower cortical layers and in callosal projection neurons. Moreover, neurons derived from individuals with TS show abnormal expression of tyrosine hydroxylase and an increase in production of norepinephrine and dopamine. These biochemical changes were reversed by treatment with roscovitine, a cyclin-dependent kinase inhibitor and an atypical L-type-channel blocker. This study provided evidence that L-type calcium channel Ca_v_1.2 is involved in the regulation of cortical neuronal differentiation in humans and offers new insights into the pathogenesis of autism in patients with TS.

### 2.2. Genetic Neurodegenerative Disorders

Genetic neurodegenerative disorders include a variety of diseases that involve the chronic and progressive loss of neuronal structure and function. To date, iPSCs have been generated from patients of many neurodegenerative disorders, including spinal muscle atrophy, familial dysautonomia, amyotrophic lateral sclerosis, Huntington's disease, Friedreich ataxia, Machado-Joseph disease, X-linked adrenoleukodystrophy, Alzheimer's disease, and Parkinson's disease (Table 2). We will comment on the current iPSC technology for these disorders in the following section and illustrate how these cell lines are helping to unravel the mechanisms of neurodegeneration.

#### 2.2.1. Spinal Muscle Atrophy

Spinal muscular atrophy (SMA) is an autosomal recessive genetic disorder caused by mutations in the survival motor neuron 1 gene (*SMN1*) that significantly reduces SMN protein expression and leads to the selective degeneration of lower *α*-motor neurons [92–95]. Although patient fibroblasts have been widely used to study SMA, motor neurons provide a better model to study the anatomy and physiology in the inherent pathology. SMA was the first neurodegenerative disease to be modeled by human iPSCs. To model SMA, Ebert et al. [96] generated iPSCs from a child with a mutation in *SMN1* and from his unaffected mother. They found that iPSCs retained the capacity to generate differentiated neural tissue and motor neurons. However, the lack of *SMN1* expression and the disease phenotype of selective motor neuron death were maintained. Interestingly, two compounds, valproic acid and tobramycin, which have been known to increase SMN levels, could partially restore the reduction in the SMN protein. Recently, Chang et al. [97] reported the establishment of five iPSC lines from the fibroblasts of an SMA patient. The authors found that neuronal cultures derived from these SMA iPSC lines exhibited a reduced capacity to form motor neurons and showed abnormal neurite outgrowth in culture. Ectopic SMN expression in these iPSC lines restored normal motor neuron differentiation and rescued the phenotype of delayed neurite outgrowth. These findings indicate that the observed abnormalities are indeed caused by the SMN deficiency and not by phenotypic diversity among iPSC lines. Taken together, these studies show that human iPSCs are useful to model the specific neuronal pathology of SMA and human iPSCs represent a promising resource to screen new drug compounds and develop new therapies for SMA [98].

#### 2.2.2. Familial Dysautonomia

Familial dysautonomia (FD) is a rare but fatal peripheral neuropathy, caused by a point mutation in the *IKBKAP* gene involved in transcriptional elongation [99–101]. FD is characterized by the depletion of autonomic and sensory neurons. The specificity to the peripheral nervous system and the mechanism of neuron loss in FD are poorly understood owing to the lack of an appropriate model system [99, 102]. Lee et al. [103] reported the derivation of patient specific FD iPSCs and the directed differentiation into cells of all three germ layers including peripheral neurons. Gene expression analysis in purified FD iPSC derived lineages demonstrated tissue-specific missplicing of *IKBKAP in vitro*. Patient-specific neural crest precursors expressed particularly low levels of the normal *IKBKAP* transcript, suggesting a mechanism for disease specificity. FD pathogenesis has been further characterized by transcriptome analysis, and cell-based assays reveal marked defects in neuronal differentiation and migration behaviour. Furthermore, FD iPSCs were used for validating the potency of candidate drugs in reversing aberrant splicing and ameliorating neuronal differentiation and migration. This study, while limited, has laid the groundwork to use reprogramming technology for modeling FD.

#### 2.2.3. Amyotrophic Lateral Sclerosis

Amyotrophic lateral sclerosis (ALS) is mainly characterized by muscular atrophy and weakness that accompanies a fast and progressive degeneration of motor neurons in the brain and spinal cord [104–109]. The majority of ALS cases are sporadic (sALS) and approximately 10% of cases are inherited (familial; fALS). More than 10 different genes have been implicated in ALS, including superoxide dismutase 1 (*SOD1*, *ALS1*), transactive response DNA-binding protein-43 (*TDP-43*, *ALS10*), fused sarcoma (*FUS*, *ALS6*), and vamp-associated protein B/C (*VAPB*, *ALS8*) [107–111]. Clinical trials based on ALS animal models have been disappointing, indicating a need for the exploration of new ALS models [104, 105, 112]. To date, three groups have successfully generated iPSCs from three different familial forms of ALS with previously identified mutations. Dimos et al. [113] generated iPSCs from a patient with a mutation in the *SOD1* gene. They found that the patient-specific iPSCs possess properties of embryonic stem cells and could be directed to differentiate into motor neurons. However, no assay of ALS related phenotypes was completed for the iPSCs derived motor neurons in this study. Mitne-Neto et al. [114] generated iPSC lines from patients with mutations in the *VAPB* gene as well as from noncarrier siblings (controls). They showed a significant reduction in the levels of VAPB protein in ALS8 iPSC derived motor neurons, suggesting that the reduction in VAPB could be involved in the pathogenesis of ALS8. They further demonstrated that the level of VAPB protein gradually increased during the differentiation of control iPSCs but not ALS8 iPSCs, suggesting that the ALS8 mutation causes a failure of VAPB protein upregulation during the induction of motor neurons. This regulation of VAPB is likely to happen at the posttranslational level since there is no difference in the mRNA levels during the differentiation between control and ALS8 iPSCs. These findings may be relevant to other forms of ALS, as the reduction in VAPB protein has been documented in motor neurons from sporadic ALS patients [115, 116]. The iPSCs that carry the *TDP-43* mutation could differentiate into neurons and functional motor neurons. The mutant neurons showed the cellular phenotypes of ALS and other TDP-43 proteinopathies, including elevated soluble and detergent-resistant TDP-43 protein levels, decreased survival, and increased vulnerability to blockade of the phosphoinositide-3-kinase (PI3K) pathway [117]. Since other cells, such as astrocytes and microglia that associate with the motor neuron niche, have been shown to play a role in the pathology of ALS [118–120], it will be interesting to see if the iPSC derived astrocytes or microglia can also recapitulate the nonneuronal aspects of the disease.

#### 2.2.4. Huntington's Disease

Huntington's disease (HD) is an autosomal dominant neurodegenerative disorder caused by an excessive expansion of a CAG trinucleotide repeat in the gene encoding the protein huntingtin, producing an elongated stretch of glutamines near the N-terminus of the protein [121–124]. The expanded repeat region causes a gain of function in the huntingtin protein, which then forms aggregates within the nucleus of certain neuronal cells [121, 122]. Many tissue culture models for Huntington's disease have been generated by a wide range of techniques. Modeling systems include nonneural human cell types (fibroblasts and lymphoblasts), immortalised or primary neurons from mice, and mouse and human ES cells [125–127]. These models can recapitulate many of the phenotypes seen in patients with HD. At this time, iPSCs have been generated from patients with HD [74, 128], transgenic HD monkeys, and mouse models [12, 129]. The human HD iPSCs have been used to generate neuronal precursors and striatal neurons. The HD iPSCs derived striatal neurons, and neuronal precursors contain the same CAG expansion as the mutation in the HD patient from whom the iPSC line was established. Moreover, the HD neural stem cells showed enhanced caspase activity upon growth factor deprivation, which is indicative of apoptosis [128]. Therefore, these differentiated cells are encouraging for a useful human HD cell model. The HD monkey iPSCs develop cellular features comparable to HD, including the accumulation of mutant huntingtin (htt) aggregates and the formation of intranuclear inclusions paralleling neural differentiation *in vitro *[129]. iPSCs from transgenic HD monkeys represent nonhuman primate modeling of human diseases. In the generation of iPSCs through somatic reprogramming of fibroblasts from the R6/2 transgenic HD mouse line, CAG expansion has no effect on reprogramming efficiency, cell proliferation rate, brain-derived neurotrophic factor levels, or neurogenic potential. In addition, these iPSCs do not show an increase in cell death either under self-renewal or differentiated conditions. However, genes that are involved in the alteration of the cholesterol biosynthesis pathway in HD are also affected in HD iPSC lines. Furthermore, one lysosomal gene is upregulated and the lysosome number is increased in HD iPSC lines [12]. One recent study further demonstrated that HD patient specific iPSCs were able to generate phenotypically normal, functional neurons *in vitro* and could survive and differentiate into neurons in the adult mouse brain after transplantation. However, astrocytes derived from these HD iPSCs showed a vacuolation phenotype, a phenomenon found in primary lymphocytes from HD patients [130]. These findings suggest that iPSCs from HD animals or patients can replicate some, but not all, of the phenotypes typically observed in the disease. In the future, modeling of other phenotypes, including the pathological changes only seen in the autopsy of patients and the electrophysiological changes seen in animal models may, become possible.

#### 2.2.5. Friedreich Ataxia

Friedreich ataxia (FRDA) is an autosomal recessive disorder characterised by neurodegeneration and cardiomyopathy [131–134]. It is caused by a trinucleotide (GAA) repeat expansion in the first intron of the *FXN* gene that results in reduced synthesis of FXN mRNA and its protein product, frataxin [135, 136]. Ku et al. [137] firstly reported the derivation of iPSCs from FRDA patient fibroblasts by transcription factor reprogramming. They found that *FXN* gene repression is maintained in the iPSCs. The GAA repeats in *FXN* in iPSCs exhibit instability similar to the patient families, where they expand and/or contract with discrete changes in length between generations. The mismatch repair enzyme MSH2, which is implicated in repeat instability in other triplet repeat diseases, is highly expressed in iPSCs and occupies the *FXN* intron 1. Knockdown of MSH2 impedes repeat expansion. This study provides a possible molecular explanation for repeat expansion in FRDA. Liu et al. [138] reported the generation of iPSC lines derived from skin fibroblasts from FRDA patients. The authors found that the patient-derived iPSC lines maintain the GAA repeat expansion and the reduced FXN mRNA expression patterns that are characteristic of the patient. Interestingly, the instability of the GAA repeat length was also found within these FRDA iPSC lines. They further demonstrated that following *in vitro* differentiation, the iPSCs can produce the two cell types primarily affected in FRDA, namely peripheral neurons and cardiomyocytes. Thus, these FRDA iPSC lines have the potentials to provide powerful tools to study the cellular pathology of FRDA.

#### 2.2.6. Machado-Joseph Disease

Machado-Joseph disease (MJD), also known as spinocerebellar ataxia type 3, is a dominantly inherited late-onset neurodegenerative disorder caused by expansion of polyglutamine (polyQ)-encoding CAG repeats in the *MJD1* (*ATXN3*) gene [139–142]. Proteolytic liberation of highly aggregation-prone polyQ fragments from the protective sequence of the *MJD1* gene product ataxin 3 (*ATXN3*) may trigger the formation of *ATXN3*-containing aggregates, the pathological hallmark of MJD [140, 141]. The levels of *ATXN3* fragments in brain tissues of MJD patients increases with disease severity, supporting a relationship between *ATXN3* processing and disease progression. The formation of early aggregation intermediates is believed to be critical for disease initiation, but the precise molecular mechanism in MJD is unknown [140, 141]. To investigate this, Koch et al. [143] generated iPSCs from MJD patients. The authors found that L-glutamate induced excitation of patient specific iPSC derived neurons initiated Ca^2+^-dependent proteolysis of *ATXN3*, followed by the formation of sodium dodecyl sulphate (SDS)-insoluble aggregates. This phenotype could be abolished by calpain inhibition, indicating a key role of calpain in *ATXN3* aggregation. They further demonstrated that aggregate formation depended on functional Na^+^ and K^+^ channels as well as ionotropic and voltage-gated Ca^2+^ channels. However, these channel effects were not observed in iPSCs, fibroblasts, or glia and hence may explain the neuron-specific phenotype of MJD. This study demonstrates the usefulness of iPSCs to investigate the aberrant protein processing associated with late-onset neurodegenerative disorders in patient-specific neurons.

#### 2.2.7. X-Linked Adrenoleukodystrophy

X-linked adrenoleukodystrophy (X-ALD) is caused by mutations in the *ABCD1* (adenosine triphosphate (ATP)-binding-cassette transporter superfamily D member 1) gene encoding a peroxisomal ATP-binding cassette (ABC) transporter, ABCD1, which is responsible for entry of long chain fatty acids (VLCFAs; C26:0 and C24:0) into peroxisomes for degradation [144–147]. With abnormally high VLCFA levels, primary manifestations occur in the nervous system, the adrenal cortex, and the Leydig cells of the testis [144, 145]. X-ALD, with an incidence of 1 in 20,000 males, shows a wide range of phenotypic variability which does not directly correlate with *ABCD1* gene mutations [146–150]. Due to the lack of an appropriate animal model system and the inaccessibility of human oligodendrocytes *in vivo* [147, 151], iPSCs may provide a unique cellular model for studying the pathology of X-ALD. Jang et al. [152] generated iPSCs from patients with the 2 major types of X-ALD, namely, childhood cerebral ALD (CCALD) and adrenomyeloneuropathy (AMN). The authors evaluated disease relevant phenotypes by pharmacological and genetic approaches. They found that both CCALD and AMN iPSCs normally differentiated into oligodendrocytes, the cell type primarily affected in the X-ALD brain, indicating no developmental defect due to the *ABCD1* mutations. Although low in X-ALD iPSCs, long chain fatty acid (VLCFA) levels were significantly increased after oligodendrocyte differentiation. VLCFA accumulation was much higher in CCALD oligodendrocytes when compared to AMN oligodendrocytes, whereas no significant difference between CCALD and AMN neurons was reported, indicating that the severe clinical manifestations in CCALD might be associated with abnormal VLCFA accumulation in oligodendrocytes. They further showed that the abnormal accumulation of VLCFA in the X-ALD oligodendrocytes can be reduced by upregulating *ABCD2* gene expression after treatment with lovastatin or 4-phenylbutyrate. Therefore, the X-ALD iPSC model recapitulates the key events of the disease process and provides a new way to understand and diagnose X-ALD disease subtypes.

#### 2.2.8. Alzheimer's Disease

AD is the most common age-related dementia, characterized by progressive memory loss and cognitive disturbances [153–157]. AD presents with a strong genetic predisposition [158, 159]. Mutations of presenilin 1 (*PS1*) and presenilin 2 (*PS2*) are causative factors for autosomal-dominant, early-onset familial AD (FAD) [158–161]. Yagi et al. [162] generated iPSCs from fibroblasts of FAD patients with mutations in *PS1* (A246E) and *PS2* (N141I) and characterized the differentiation of these cells into neurons. They found that FAD iPSC derived differentiated neurons have increased amyloid *β*42 secretion, recapitulating the molecular pathogenesis of mutant presenilins. Amyloid *β*42 secretion from the neurons sharply responded to *γ*-secretase inhibitors and modulators, indicating the potential for the identification and validation of candidate drugs. This study demonstrates that the FAD iPSC derived neurons could be an effective model of AD and provides an innovative strategy for the study of age-related neurodegenerative diseases. More recently, Israel et al. [163] reprogrammed fibroblasts from patients with FAD caused by a duplication of the *APP* gene (termed *APP *(Dp)) into iPSC lines. Compared to controls, iPSC-derived purified neurons from the *APP* (Dp) patients exhibited significantly higher levels of the pathological markers amyloid *β*40, phospho-tau (Thr 231) and active glycogen synthase kinase-3*β* (aGSK-3*β*), but all cells exhibited normal electrophysiological activity. Neurons from *APP* (Dp) also accumulated large RAB5-positive early endosomes compared to controls. Treatment of purified neurons with *β*-secretase inhibitors, but not *γ*-secretase inhibitors, reduced the phospho-Tau (Thr 231,) and aGSK-3*β* levels. These results suggest a direct role of APP proteolytic processing, but not amyloid *β*, in GSK-3*β* activation and tau phosphorylation in human neurons. More recently, Koch et al. [164] demonstrated that neurons derived from iPSCs with the *PS1 *(L166P) mutation showed a partial loss of *γ*-secretase function, which results in the decreased production of amyloid *β*40 and an increased amyloid *β* 42/40 ratio. These neurons are also resistant to *γ*-secretase modulation by nonsteroidal anti-inflammatory drugs (NSAIDs). The patient-specific iPSCs thus provide a human neuronal system to study AD pathogenesis and to screen compounds for the pharmaceutical treatment of AD.

#### 2.2.9. Parkinson's Disease

Parkinson's disease (PD) is the most common neurodegenerative movement disorder. It is due to the progressive degeneration of the dopaminergic (DA) neurons in the substantia nigra and is accompanied by the appearance of intraneuronal inclusions enriched in alpha-synuclein called Lewy bodies [165, 166]. It is becoming increasingly clear that genetic factors contribute to the complex pathogenesis of PD. Genes including *PARK2*, *SNCA*, *PARKIN*, *PINK1*, *DJ-1*, *UCHL1*, *LRRK2*, *PARK7*, *GBA*, *SNCAIP,* and *ATP13A2* have been found to be directly associated with Parkinson's disease [166–169]. The iPSCs have been successfully generated from PD patients with mutations in some of these genes [170–175].

Nguyen et al. [171] generated iPSCs that carry the mutation in the *leucine-rich repeat kinase-2* (*LRRK2*) gene and differentiated the cells into DA neurons. The high penetrance of the *LRRK2* mutation and its clinical resemblance to sporadic PD was observed in this process, suggesting that iPSCs could serve as a platform for studying PD. The authors found that the expression of key oxidative stress-response genes and the *α*-synuclein protein was increased in the DA neurons derived from PD iPSCs. The mutant DA neurons were also more sensitive to caspase-3 activation and cell death when exposed to stress agents. This enhanced stress sensitivity is consistent with early phenotypes of PD and may become a potential therapeutic target for this disorder. Recently, Sánchez-Danés et al. [175] reported that DA neurons differentiated from PD iPSCs with the *LRRK2* mutation showed morphological alterations, such as reduced numbers of neurites and neurite arborization, as well as accumulation of autophagic vacuoles. These morphological alterations could be greatly exacerbated by further induction of autophagy and/or inhibition of lysosomal proteolysis, indicating autophagic compromise in DA neurons from PD iPSCs, which occurs at the level of autophagosome clearance.

Seibler et al. [172] reported the generation of iPSCs from skin fibroblasts of PD patients with nonsense or missense mutations in the *PTEN-induced putative kinase 1* (*PINK1*) gene. When differentiated into DA neurons and processed for mitochondrial depolarization, the cells showed impaired recruitment of lentivirally expressed parkin to the mitochondria, increased mitochondrial copy number, and upregulation of peroxisome proliferator-*γ* (PPAR*γ*) coactivator-1*α* (PGC-1*α*), an important regulator of mitochondrial biogenesis. Importantly, these alterations were corrected by the lentiviral expression of wild-type *PINK1* in mutant iPSC derived *PINK1* neurons. This study indicates that fibroblasts from genetic PD can be reprogrammed and differentiated into DA neurons. These iPSC derived neurons exhibit distinct phenotypes that should be amenable to further studies of molecular and cellular mechanisms of PD. Triplication of *SNCA*, encoding *α*-synuclein, causes a fully penetrant, aggressive form of PD with dementia. The *α*-synuclein dysfunction is the critical pathogenic event in Parkinson's disease that leads to multiple system atrophy and dementia with Lewy bodies [167, 169]. Devine et al.[170] produced multiple iPSC lines from a SNCA triplication patient and from an unaffected first-degree relative. They found that DA neurons differentiated from the iPSCs of the patient produced double the amount of *α*-synuclein protein in comparison to the neurons from the unaffected relative, thus precisely recapitulating the primary cause of PD in these individuals. This model represents an exceptional experimental system to screen compounds that reduce the levels of *α*-synuclein and to investigate the cellular mechanisms of neurodegeneration caused by *α*-synuclein dysfunction. The lack of a phenotype in parkin knockout mice suggests that the human neuronal system is needed to model the disease. Jiang et al. [174] demonstrated that in DA neurons from iPSCs of PD patients with parkin mutations, the transcription of monoamine oxidases and oxidative stress are greatly increased, DA uptake is reduced, and spontaneous DA release is increased. Lentiviral expression of parkin, but not the PD related mutant, rescues these phenotypes, suggesting that parkin controls dopamine utilization in DA neurons by modulating DA neurotransmission and suppressing dopamine oxidation. This study thus provides additional targets for screening pharmaceutical therapies of PD.

One obvious limitation in the application of iPSC technology is the inability to perform experiments under genetically defined conditions. This is especially crucial to late age onset disorders in which the *in vitro* phenotypes are usually subtle and susceptible to effects of genetic background variations. To solve this problem, Soldner et al. [173] combined zinc finger nuclease (ZFN) mediated genome editing and iPSC technology. They generated sets of isogenic disease and control human pluripotent stem cells that differ exclusively at either of two susceptibility variants for PD by modifying the point mutations in the *α*-synuclein gene. This approach to genetically correct disease-causing point mutations in patient derived iPSCs represents significant progress in basic biomedical research and an advance for iPSC technology.

### 2.3. Concerns in Modeling Neurogenetic Disorder by iPSCs

Although iPSCs are promising for modeling neurogenetic disorders, there are still limitations. In most cases of neurogenetic disorders, we need to determine whether typical traits of neurogenetic disorders can be observed in the context of the iPSC models. At the molecular level, such as disorder associated protein expression or global gene expression levels, it is likely that patient iPSC derived neuronal cultures will recapitulate phenotypes of the disorders. But for late-onset neurodegenerative diseases, the patient iPSC derived neurons may not be able to show typical patient brain pathology, such as Lewy bodies in PD [5, 19, 176, 177].

On the other hand, it is also very important to reproduce or validate data derived from patient-specific iPSC lines, given the substantial phenotypic diversity of these cell lines and the genetic heterogeneity of the patients. Notably, suppression of patient iPSC culture associated phenotypes by repair of the relative genetic defects could further validate these models. Despite all these concerns, previous studies have already provided strong evidence for iPSC technology as a powerful approach to model neurogenetic disease [5, 19, 176, 177].

## 3. iPSC Based Therapeutic Strategies for Neurogenetic Disorders

### 3.1. Drug Screening and Development

iPSC technology provides a platform for the discovery of novel bioactive compounds through molecular dissection of the pathological process [5]. The inspiring examples to demonstrate the potential of iPSCs in screening drug candidates arise from the above mentioned studies in modeling neurogenetic disorders, such as RTT [27], TS [91], SMA [96, 97], FD [102, 103], MJD [143], X-ALD [152], AD [162, 163], and PD [170–172]. Currently, iPSCs have been shown to be valuable for testing small numbers of compounds for efficacy and toxicity in a specific patient or population of patients.

It is clear that iPSC technology can be a useful approach for determining which drugs or drug combinations are effective in humans or in specific patients [5, 16, 19, 102]. To make this technology more powerful, it is essential and crucial to validate the molecular and cellular phenotypes identified in iPSC derived neurons or glial cells. High-throughput drug screening and development requires uniform populations of neurons or glial cells. With the development of iPSC technology, iPSC derived neurons or glial cells will be generated in larger quantities with higher uniformity.

### 3.2. Cell Therapy

The generation of iPSCs from patients with neurogenetic disorders permits the production of large numbers of CNS cells with the patients' exact genotype. These cells are immune matched to the individual patient—a long desired goal of regenerative medicine. iPSCs provide autologous cell sources for cell replacement/neuroprotection strategies in patients with neurodegenerative diseases. Promising results have been reported with rodent and human iPSC derived neurons such as the improvement of the behavioural symptoms in the rat model of PD [178, 179]. In addition to the transplantation of specific neurons from iPSCs for replacement therapy, transplantation of glial cells from iPSCs can also be used for neuroprotection [180]. In patients with spinal motor neuron diseases, the problem of replacing motor neurons seems daunting, considering that these cells must extend and correctly innervate specific CNS areas. Transplanting cells as therapeutic support cells, rather than as replacement neurons, is an additional and potentially alternative mode of cell therapy for motor neuron diseases [5, 176].

The iPSC technology has largely circumvented political and ethical hurdles previously associated with human embryonic stem cell research. However, several major challenges must be overcome before cell therapy using iPSC technology can be applied clinically [5]. First, among many other safety issues, the risk of cancer must be resolved [5]. iPSC derived neurons and glial cells will not be suitable for transplantation until the oncogenic genes and retroviruses used are replaced with more controlled cell reprogramming [19, 176]. New strategies to reprogram cells in the absence of integrating viral vectors have been reported [181–183] in addition to more efficient integrative approaches [184, 185]. Second, differentiating iPSCs to the specific type(s) of required CNS cells or devising accurate methods to purify the desired cells are key priorities. Building on the progresses that have already been made using stem cells, researchers should continue to improve the understanding of directed differentiation and to develop new protocols. These protocols will bring iPSC technology one step closer to patient-matched cells or tissues for clinical transplantation. Third, it should be necessary to understand and correct any genetic defects in the patient's neurons and glial cells before they can be rationally used for cell therapy. A major concern here is that iPSC derived therapeutics may recapitulate the patient's disease process, due to their genetic propensity. In the context of single-gene disorders, such changes may theoretically be genetically repaired *in vitro* prior to transplantation [176]. For neurodegenerative disorders, such as PD, the relatively brief lifetime of the reprogrammed cells may sufficiently delay an intrinsic pathogenic program. However, cell extrinsic factors in the host patient CNS environment may promote pathogenesis in therapeutic transplanted cells [186]. If the goal is to actually repair the neural circuit, then the most significant hurdle will be the regrowth of projections to the proper target structure in a manner that respects the organization of the neural network. The mechanical barrier caused by disease or injury related gliosis may also affect the restoration of damaged neuronal networks in the adult CNS [5]. These represent some, but certainly not the only pressing issues that must be overcome before cell therapy can be translated in efficacious ways to the clinic. Despite all these potential challenges, iPSC technology, although nascent, represents a remarkable progress toward cell therapy for neurological disorders.

## 4. Conclusions

Given all the limitations and disadvantages discussed above, we must realize that iPSC derived modeling systems are only one tool in the array of approaches needed to understand and treat human neurogenetic disorders. In consideration of iPSC models for the advancement of therapy for these disorders, we must also understand the pathophysiology at the systems level. A full understanding will require the balancing and integration of information at multiple levels [177, 187]. The data derived from primary CNS cells, human iPSC, or other stem cell derived CNS cells, transgenic animal models, and human studies must be integrated into the broader landscape. In this landscape, each level of information will be able to inform the other to enhance our understanding as we move forward. The iPSCs fill a critical gap in our experimental approaches by providing live, functioning human CNS cells with the genetic backgrounds of patients. They provide an essential link between animal model studies and the assessment of human postmortem brain tissue and live brain functioning. Studies of human CNS cells derived from the iPSCs of patients will give us valuable insights into the mechanisms of pathogenesis and future iPSC research will facilitate drug discovery, cell therapy, and new modes of diagnosis for neurogenetic disorders [3, 19, 177].

## Figures and Tables

**Table 1:** Genetic neurodevelopmental disorders modeled with iPSCs.

Disease	Genetic defects	iPSC derived cell types	Disease phenocopied in iPSCs or differentiated cells	Drug or functional tests
Rett syndrome	Mutation in *MECP2 *	Neurons; glutamatergic neurons	Yes	Yes
	Mutation in *CDKL5 *	Neurons	NA	No
Fragile X syndrome	CGG triplet repeat expansion resulting in the silencing of *FMR1 *	Neurons and glia	Yes	No
Down syndrome	Trisomy 21	Cortical neurons	Yes	Yes
Angelman syndrome	Lack of *UBE3A *expression due to genomic imprinting	Neurons	Yes	Yes
Prader-Willi syndrome	Lack of expression of genes in paternal chromosome region 15q11-q13 due to genomic imprinting	Tissues of the three germ layers, including neurons	NA	No
Timothy syndrome	Mutation in the L-type calcium channel Ca_v_1.2	Neurons	Yes	Yes

**Table 2:** Genetic neurodegenerative disorders modeled with iPSCs.

Disease	Genetic defects	iPSC derived cell types	Disease phenocopied in iPSCs or differentiated cells	Drug or functional tests
Spinal muscular atrophy	Mutation in *SMN1 *	Neuronal cultures; motor neurons	Yes	Yes
Familial dysautonomia	Mutation in *IKBKAP *	Cells of all three germ layers including peripheral neurons	Yes	Yes
Amyotrophic lateral sclerosis	Mutation in *SOD1 *	Motor neurons	NA	No
	Mutation in *VAPB *	Motor neurons	Yes	No
	Mutation in *TAP-43 *	Neurons and motor neurons	Yes	Yes
Huntington's disease	Excessive expansion of CAG repeat in Huntingtin gene	Neuronal precursors, striatal neurons, astrocytes	Yes	Yes
Friedreich ataxia	GAA repeat expansion in the *FXN* gene	Peripheral neurons and cardiomyocytes	Yes	No
Machado-Joseph disease	Expansion of CAG repeats in the *MJD1* (*ATXN3*) gene	Neurons, fibroblasts and glia	Yes	Yes
X-linked adrenoleuko-dystrophy	Mutation in *ABCD1 *	Oligodendrocytes and neurons	Yes	Yes
Alzheimer's disease	Mutations in *PS1* and *PS2 *	Neurons	Yes	Yes
	Duplication of *APP *	Neurons	Yes	Yes
Parkinson's disease	Mutation in *LRRK2 *	Dopaminergic neurons	Yes	Yes
	Mutations in *PINK1 *	Dopaminergic neurons	Yes	Yes
	Triplication of *SNCA *	Dopaminergic neurons	Yes	Yes
	Mutation in *PARKIN *	Dopaminergic neurons	Yes	Yes
